# Scurvy incidence trend among children hospitalised in France, 2015–2023: a population-based interrupted time-series analysis

**DOI:** 10.1016/j.lanepe.2024.101159

**Published:** 2024-12-06

**Authors:** Zein Assad, Maelle Trad, Zaba Valtuille, Cécile Dumaine, Albert Faye, Tania Ikowsky, Florentia Kaguelidou, Lindsay Osei, Naim Ouldali, Ulrich Meinzer

**Affiliations:** a Department of General Paediatrics, Paediatric Internal Medicine, Rheumatology and Infectious Diseases, National Reference Centre for Rare Paediatric Inflammatory Rheumatisms and Systemic Autoimmune Diseases (RAISE), Robert-Debré University Hospital, Assistance Publique-Hôpitaux de Paris, F-75019, Paris, France; b Department of Paediatrics, Cayenne Hospital, F-97300, Cayenne, French Guiana, France; c Université Paris Cité, INSERM, Infection, Antimicrobials, Modelling, Evolution (IAME) Research Unit, UMR 1137, F-75018 Paris, France; d Center of Clinical Investigations, INSERM CIC 1426, Robert-Debré University Hospital, Assistance Publique-Hôpitaux de Paris, F-75019, Paris, France; e Université Paris Cité, INSERM, Épidémiologie Clinique-Évaluation Économique appliqué aux populations Vulnérables (ECEVE), UMR 1123, F-75010, Paris, France; f Université Paris Cité, INSERM, Perinatal and Paediatric Pharmacology and Therapeutic Assessment, UMR 1343, F-75019, Paris, France; g Centre for Clinical Investigations Antilles Guyane, INSERM CIC 1424, Cayenne Hospital, F-97300, Cayenne, French Guiana, France; h Université Paris Cité, INSERM, Centre de Recherche sur l’Inflammation (CRI), UMR 1149, F-75018, Paris, France

**Keywords:** Scurvy, Malnutrition, Food insecurity, COVID-19 pandemic, Nutritional deficiencies

## Abstract

**Background:**

Scurvy, historically rare in-high income countries, has re-emerged as an indicator of socioeconomic and dietary disparities. Limited data exist on scurvy trends among European children, particularly following socioeconomic changes since the COVID-19 pandemic. This study analysed scurvy incidence trends among French children over a nine-year period, examining potential post-pandemic increases.

**Methods:**

This cohort study used an interrupted time-series analysis of patient records from a national hospital-based French surveillance system. All children aged <18 years hospitalized with scurvy and malnutrition from January 2015 to November 2023 were included. The monthly incidence of hospitalized scurvy per 100,000 children was analysed using a segmented linear regression model with autoregressive error. Incidence of hospitalization for malnutrition was analysed as secondary outcome and for urinary tract infection and vitamin D deficiency as control outcomes.

**Findings:**

A total of 888 children were hospitalized with scurvy (median age, 11 years; interquartile range [IQR], 4–15; 431 boys [48.5%]). The COVID-19 pandemic in March 2020 was associated with a significant increase in scurvy incidence (cumulative increase, 34.5%; 95% confidence interval [CI], 12.7–56.3; p = 0.002) and severe malnutrition (cumulative increase, 20.3%; 95% CI, 10.7–29.9; p < 0.001). The increased incidence of scurvy was correlated with the rise in the consumer price index. In contrast, no change was found for the two control outcomes.

**Interpretation:**

This study identifies a significant increase in scurvy and severe malnutrition post-COVID-19, associated with inflation and socioeconomic instability, emphasizing the urgent need for targeted nutritional support for at-risk paediatric populations.

**Funding:**

None.

**Research in context**

**Evidence before this study**

Scurvy has historically been rare in high-income countries due to improved nutrition. We searched PubMed for articles published in English from January 1, 2014, to August 30, 2024, using the terms “scurvy” and “children” or “paediatric”. We found approximately 40 studies which were generally limited to reviews and case series. A few alerts raised concerns about a resurgence of scurvy, exacerbated by the socioeconomic impact of the COVID-19 pandemic and subsequent multiple crises. Nonetheless, comprehensive data on long-term trends in scurvy in high-income countries, particularly in Europe, are lacking.

**Added value of this study**

This study provides the first large-scale longitudinal analysis of the incidence of hospitalised scurvy in French children over a nine-year period, indicating a significant increase in cases associated with the COVID-19 pandemic, together with an increase in severe malnutrition. The increased incidence of scurvy and severe malnutrition was correlated with the consumer price index, an indicator of inflation.

**Implications of all the available evidence**

The observed increase in scurvy and severe malnutrition since the COVID-19 pandemic highlights the need for targeted nutrition interventions and increased awareness among health care providers and policymakers to address emerging nutritional deficiencies in high-income countries.

## Introduction

Scurvy is a serious condition resulting from a deficiency of vitamin C, also known as ascorbic acid.^1^^,^^2^ Vitamin C is an essential water-soluble nutrient that plays a critical role in the biosynthesis and maintenance of collagen, a structural protein vital for the integrity of skin, blood vessels, bones and other connective tissues, and in various aspects of the immune response.^3^^,^^4^ Because humans cannot synthesize vitamin C endogenously, sufficient dietary intake is required to ensure proper collagen formation and to prevent systemic diseases. Main food sources of vitamin C include citrus fruits, potatoes, spinach, strawberries, cauliflower, and broccoli. For infants, breast milk typically provides sufficient vitamin C, provided it is not heated or pasteurised.^5^ When vitamin C intake is deficient (<10 mg/day), scurvy can develop rapidly, often within 1–3 months.^4^^,^^6^ Deficiencies in vitamin C disrupt collagen synthesis and lead to the development of scurvy, which is characterized by impaired wound healing, mucocutaneous bleeding due to vessel leakage, corkscrew hairs, bone pain, among other clinical manifestations.^1^^,^^2^

While scurvy has long been considered a rare and outdated condition, except under specific settings of displaced populations or refugee camps, due to increased awareness of the importance of diet and nutrition, recent years have seen a rise in sporadic cases, even in high-income countries.^1^^,^^2^^,^^7^ Currently, scurvy tends to occur among at-risk individuals,^8^ particularly children from low-income households with nutritional deficiencies arising from biological, psychological, or environmental factors.^2^ Comprehensive data on the long-term trend of scurvy among children in European countries are currently scarce.

The COVID-19 pandemic had a major psychological and socio-economic impact and widened social inequalities,^9^^,^^10^ and the major socio-geopolitical conflicts, such as the war in Ukraine, that followed the COVID-19 period have exacerbated them. There is growing concern that the socio-economic challenges posed by the pandemic have increased the cost of food, limiting access to fresh and varied food and potentially leading to increased rates of scurvy and malnutrition.^11^, ^12^, ^13^ However, the association between socio-economic disparities linked to the COVID-19 pandemic and the potential increase in the incidence of scurvy in children has not been studied in depth.

The primary objective of this study was to examine the incidence of scurvy in French children over a 9-year period and to determine whether the incidence has increased since the onset of the COVID-19 pandemic. The secondary objective was to evaluate the incidence of both severe and mild malnutrition during the same timeframe.

## Methods

### Study design

We conducted a population-based interrupted time-series analysis of patient data from a hospital-based French national surveillance system over nine years (January 1, 2015–November 31, 2023). Access to the French Medicalization of Information Systems Program (Programme de Médicalisation des Systèmes d'Information [PMSI]) was requested from and approved by the National Commission on Information and Liberty. As part of an ongoing continuous mission of public health using anonymous aggregated data for public health purposes, this study did not require ethical committee approval or written informed consent. The Strengthening the Reporting of Observational Studies in Epidemiology (STROBE) guidelines were followed to report this study.^14^

### Study data and settings

The data were obtained from the PMSI, which is a comprehensive national database that contains all hospital discharge records in France, as previously described.^15^ Diagnoses related to the hospitalizations were recorded according to the International Statistical Classification of Diseases, Tenth Revision (ICD-10), and following a national guideline for the coding of each diagnosis (Technical Agency for Hospital Information [ATIH]).^16^

### Inclusion criteria and data collection

All children aged less than 18 years hospitalized with scurvy in France from January 2015 to November 2023 were included. Scurvy was defined as ICD-10 discharge diagnosis code E54. Malnutrition, including severe protein-calorie malnutrition (code E43) and protein-calorie malnutrition of mild and moderate degree (codes E44, E440, E441, E46) malnutrition, vitamin D deficiency (codes E55, E550, E559), and iron deficiency (codes E611, D509) were also examined.

The following data were collected for each patient: age, sex, underlying conditions such as autistic disorder, dates and length of hospitalization, hospital death, and coverage by State Universal Health Insurance. In France, eligibility for State Universal Health Insurance (Couverture Maladie Universelle [CMU]) is based on income thresholds, residency requirements, citizenship, and age criteria.^17^ This information was therefore used as a proxy for low socio-economic status.^18^ To calculate the incidence of new cases per 100 000 children, we used the age-specific French population demographics obtained from the National Institute for Statistics and Economic Studies as the denominator.^19^

### Study periods

We organized the study into two periods according to the beginning of the COVID-19 pandemic: (1) The pre-COVID-19 period, from January 2015 to March 2020; (2) the post-COVID period, from April 2020 to November 2023.

### Outcome and measures

The primary outcome was the monthly incidence of hospitalized scurvy per 100,000 children aged less than 18 years. The secondary outcomes were (1) the incidence of scurvy and malnutrition by age group, (2) by sex, (3) the incidence of malnutrition by severity (severe vs. mild and moderate), (4) the percentage of included children covered by State Universal Health Insurance by study period, and (5) the correlation of the incidence of scurvy, malnutrition, and iron deficiency with the consumer price index. The latter is an instrument for measuring inflation, estimating the average price variation of goods consumed by households between two given periods. This index, updated annually, is based on the observation of a fixed basket of goods and services, with each product weighted in the overall index according to its share in household consumption expenditure.^20^

We analysed two control outcomes that were not expected to be influenced by the COVID-19 pandemic: the monthly incidence of urinary tract infection and vitamin D deficiency per 100,000 children aged less than 18 years over the same period.

### Statistical analysis

The change in the incidence of scurvy associated with the COVID-19 pandemic was estimated by the means of a segmented linear regression model with autoregressive error.^21^ Seasonality was accounted for using an additive model. We used an autoregressive–moving-average term to account for the remaining autocorrelation. The time unit was 1 month.

We hypothesized that the COVID-19 pandemic would have a gradual impact on the incidence of scurvy, assuming restricted access to fresh and diverse foods developed progressively, combined with the delay between the reduced nutritional intake and the onset of the disease. Thus, we included time as a continuous variable to estimate the trend in the pre-COVID-19 period (pre-intervention) and another continuous variable counting the months in the post-COVID-19 period (from March 2020) to estimate the change of slope following COVID-19. The model was used to generate a counterfactual scenario assuming the COVID-19 pandemic had not taken place, based on disease data from the 5 pre-pandemic years. The estimated cumulative change in incidence was expressed as the percentage change between the incidence fitted by the model and the estimated counterfactual incidence, which was calculated for each time point of the post-COVID-19 period. The validity of the segmented regression model was assessed by visual inspection of the correlograms (autocorrelation and partial autocorrelation functions) and residuals analysis. We checked whether the residuals of the models were normally distributed and showed constant variance over time.^22^

We performed three sensitivity analyses to assess the robustness of the study findings: (1) a segmented linear regression model with seasonality accounted for by harmonic terms (sines and cosines) with 6- and 12-month periods, and (2) with 3-, 6-, and 12-month periods (instead of 12-month periods in the main analysis) to explore potential non-yearly seasonal patterns; and (3) a segmented linear regression model including a transitional period of one year (March 2020 to March 2021) to account for the potential delay between the onset of the COVID-19 pandemic and its subsequent impact on the incidence of scurvy.

The correlation between the incidence of scurvy and the consumer price index over the study period was analysed using the nonparametric Spearman correlation method. Finally, the distribution of the percentage of patients covered by Universal Health Insurance between the two study periods was compared using the Student's t-test or the nonparametric Mann–Whitney test.

All the statistical tests were two-sided, and a p value of less than 0.05 was considered to indicate statistical significance. The data were extracted from the PMSI database using SAS (SAS Institute) and statistical analyses were performed using R version 4.4.0 (R Project for Statistical Computing).

### Role of the funding source

No funding was received for this work.

## Results

### Characteristics of patients with scurvy

From January 2015 to November 2023, 888 children (median age, 11 years; interquartile range [IQR], 4–15 years; 431 boys [48.5%]) were hospitalized with scurvy in France. The median length of hospital stay for these patients was 4 days (IQR 0–12). The number of hospitalized children for each diagnosis, by age group and study period, as well as patient characteristics are presented in Table 1.

**Table 1:** Baseline characteristics of hospitalized children, January 2015–November 2023.

Characteristic	Pre-COVID period^a^	Post-COVID period^b^	Total
**Scurvy**			
Number of cases	352	536	888
Male, No. (%)	163 (46.3)	268 (50)	431 (48.5)
Female, No. (%)	189 (53.7)	268 (50)	457 (51.5)
Age group, No. (%)	NA = 1	NA = 4	NA = 5
≤4 y	87 (24.8)	131 (24.6)	218 (24.7)
5–10 y	47 (13.4)	163 (30.6)	210 (23.8)
11–17 y	217 (61.8)	238 (44.7)	455 (51.5)
Malnutrition, No. (%)	87 (24.7)	160 (29.9)	247 (27.8)
Autistic disorder, No. (%)	7 (2.0)	31 (5.8)	38 (4.3)
Length of stay, median (IQR), d	5 (0–12.3)	4 (0–11)	4 (0–12)
Death, No. (%)	1 (0.28)	3 (0.56)	4 (0.45)
**Malnutrition**			
Number of cases	74,278	61,082	135,360
Severe, No. (%)	27,115 (36.5)	32,250 (52.8)	59,365 (43.9)
Mild and moderate, No. (%)	47,163 (63.5)	28,832 (47.2)	75,995 (56.1)
Male, No. (%)	35,465 (47.7)	23,766 (38.9)	59,231 (43.8)
Female, No. (%)	38,813 (52.3)	37,316 (61.1)	76,129 (56.2)
Age group, No. (%)	NA = 870	NA = 1031	NA = 1901
≤4 y	28,768 (39.2)	17,525 (29.2)	46,293 (34.7)
5–10 y	13,331 (18.2)	8318 (13.9)	21,649 (16.2)
11–17 y	31,309 (42.7)	34,208 (57.0)	65,517 (49.1)
Autistic disorder, No. (%)	198 (0.27)	216 (0.35)	414 (0.31)
Length of stay, median (IQR), d	4 (1–13)	4 (0–12)	4 (0–12)
Death, No. (%)	647 (0.87)	412 (0.67)	1059 (0.78)
**Control outcomes**			
**Vitamin D deficiency**			
Number of cases	20,843	23,160	44,003
**Urinary tract infection**			
Number of cases	116,552	67,822	184,374

Abbreviation: IQR, interquartile range.

a Pre-COVID-19 period from January 2015 to March 2020.

b Post-COVID-19 period from April 2020 to November 2023.

### Association of the COVID-19 pandemic with the incidence of scurvy

**Fig. 1:**
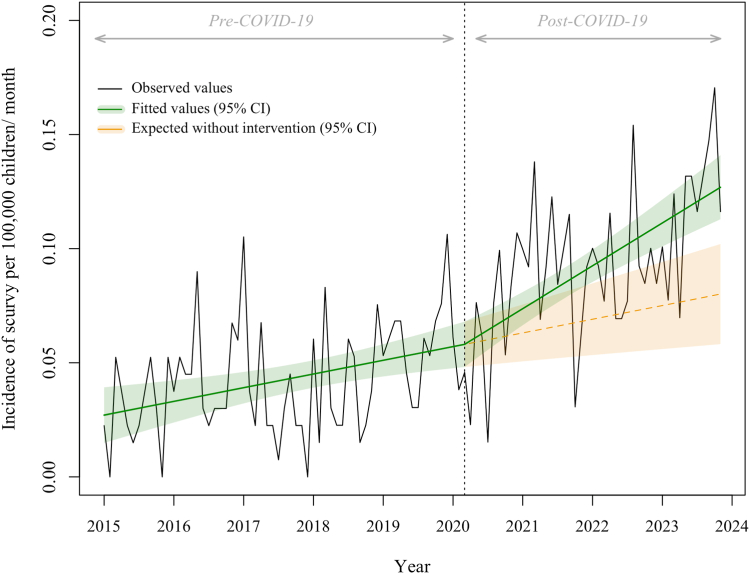
**Association of the COVID-19 pandemic with the monthly incidence of Scurvy per 100,000 children younger than 18 years (N = 888)**. The black line shows the observed data. The green line shows the model estimates based on observed data using the segmented linear regression model. The orange dotted line shows the expected values of the scenario without the COVID-19 pandemic, using the same model. The green and orange shadings indicate the 95% confidence intervals. The dotted vertical line indicates the COVID-19 pandemic in March 2020. Abbreviation: CI, confidence interval.

During the pre-COVID-19 period (2015–2020), we observed a significant steady increase in the incidence of scurvy. This increase was estimated to 0.05% per month (95% confidence interval [CI], 0.02 to 0.08). The COVID-19 pandemic was associated with a significant change in the incidence of scurvy (change of slope, 1.9% per month; 95% CI, 0.68–3.0), with an estimated cumulative increase of 34.5% (95% CI, 12.7–56.3; p = 0.002) by the end of the study (Fig. 1, Table 2). This surge was most pronounced among children aged 5–10 years, with a 200.8% cumulative increase (95% CI, 132.7–268.9; p < 0.001), and among girls (cumulative increase, 65.8%; 95% CI, 30.7–101.0; p < 0.001) (Table 2 and eFigure S1). The quality assessment of the main segmented linear regression model was satisfactory (eFigure S2 and eTable S1 in the Supplement). The three sensitivity analyses provided similar results, including the model with a one-year transitional period to account for the potential delay between the onset of the COVID-19 pandemic and the change in the incidence of scurvy (eTable S2 and eFigures S3–S6 in the Supplement).

**Table 2:** Association of the COVID-19 pandemic with the incidence of scurvy in France, January 2015–November 2023.

Outcome	Monthly change in trend in the pre-COVID-19 period^a^, % (95% CI)	Change of slope following COVID-19^b^, % (95% CI)	Cumulative change in the post-COVID-19 period^c^, % (95% CI)	p value for cumulative change
**Main outcome**^d^				
Incidence of scurvy^e^	0.05 (0.02–0.08)	1.9 (0.68–3.0)	34.5 (12.7–56.3)	0.002
**Control outcomes**^d^				
Incidence of vitamin D deficiency^e^	2.7 (2.0–3.4)	0.18 (−0.29 to 0.64)	3.4 (−5.4 to 12.1)	0.45
Incidence of urinary tract infection^e^	−2.5 (−3.3 to −1.7)	−0.08 (−0.22 to 0.06)	−1.7 (−4.9 to 1.4)	0.28
**Secondary outcomes**^d^				
Incidence of scurvy by sex^e^				
Male	0.08 (0.03–0.12)	0.74 (−0.97 to 2.5)	13.0 (−17.1 to 43.1)	0.40
Female	0.02 (−0.02 to 0.06)	3.2 (1.5–5.0)	65.8 (30.7–101.0)	<0.001
Incidence of scurvy by age group,^e^ y				
≤4	0.1 (0.04–0.16)	0.59 (−1.2 to 2.3)	10.2 (−20.3 to 40.7)	0.51
5–10	0.02 (−0.02 to 0.05)	10.7 (7.1–14.4)	200.8 (132.7–268.9)	<0.001
11–17	0.06 (−0.001 to 0.12)	0.38 (−1.1 to 1.9)	7.3 (−22.1 to 36.7)	0.63
Incidence of severe malnutrition^e^	2.4 (1.5–3.3)	1.0 (0.54–1.5)	20.3 (10.7–29.9)	0.001
By sex				
Male	1.2 (0.69–1.7)	0.18 (−0.17 to 0.53)	3.7 (−3.6 to 10.9)	0.32
Female	3.7 (2.2–5.1)	1.6 (0.90–2.2)	29.9 (17.3–42.6)	<0.001
By age group, y				
≤4	2.3 (1.4–3.1)	0.05 (−0.30 to 0.40)	1.0 (−6.2 to 8.3)	0.78
5–10	0.42 (0.08–0.75)	0.12 (−0.36 to 0.59)	2.5 (−7.6 to 12.6)	0.63
11–17	4.9 (2.9–6.9)	1.6 (0.88–2.3)	30.5 (16.9–44.2)	<0.001
Incidence of mild and moderate malnutrition^e^	0.47 (−0.42 to 1.4)	−0.51 (−0.85 to −0.16)	−11.2 (−18.7 to −3.6)	0.005
By sex				
Male	−0.40 (−1.4 to 0.62)	−0.39 (−0.80 to 0.01)	−9.0 (−18.3 to 0.33)	0.06
Female	1.3 (0.37–2.3)	−0.61 (−0.97 to −0.24)	−13.0 (−20.7 to −3.7)	0.001
By age group, y				
≤4	0.34 (−1.6 to 2.3)	−0.56 (−0.95 to −0.17)	−12.6 (−21.1 to −3.9)	0.005
5–10	−0.56 (−1.2 to 0.06)	−0.15 (−0.67 to 0.36)	−3.6 (−15.7 to 8.4)	0.56
11–17	1.9 (0.75–3.1)	−0.62 (−1.0 to −0.21)	−13.1 (−21.7 to −4.5)	0.004
Incidence of overall malnutrition^e^	2.8 (1.4–4.3)	0.13 (−0.20 to 0.47)	2.8 (−9.8 to 4.2)	0.44
By sex				
Male	0.73 (−0.53 to 2.0)	−0.19 (−0.52 to 0.15)	−4.1 (−11.4 to 3.2)	0.27
Female	4.9 (2.9–7.0)	0.39 (−0.03 to 0.81)	7.9 (−0.71 to 16.4)	0.08
By age group, y				
≤4	2.5 (0.28–4.7)	−0.36 (−0.67 to −0.06)	−7.9 (−14.5 to −1.2)	0.02
5–10	−0.18 (−1.0 to 0.64)	−0.04 (−0.47 to 0.40)	−0.80 (−10.7 to 9.2)	0.88
11–17	6.7 (3.9–9.4)	0.49 (−0.004 to 0.98)	9.8 (−0.08 to 19.6)	0.05

Abbreviation: CI, confidence interval.

a Pre-COVID-19 period from January 2015 to March 2020.

b COVID-19 pandemic started in March 2020.

c Post-COVID-19 period from April 2020 to November 2023.

d Analysis by segmented linear regression model.

e Monthly incidence expressed as the number of cases per 100,000 children per month.

### Incidence of malnutrition

**Fig. 2:**
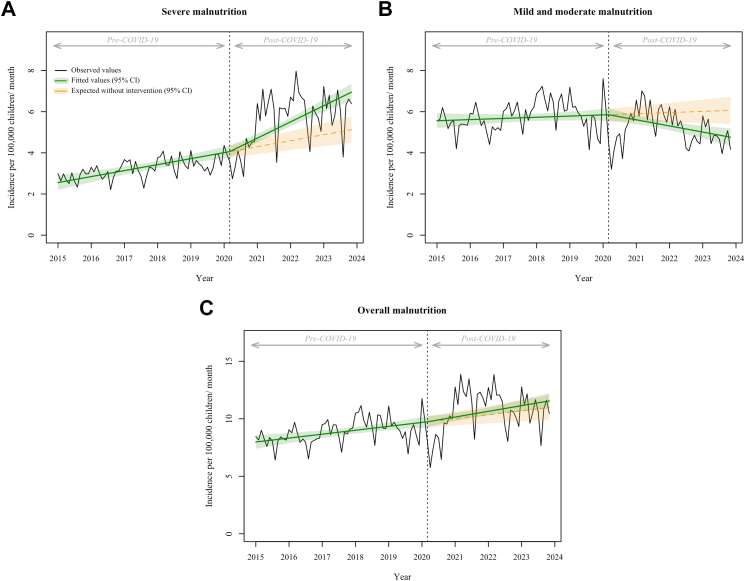
**Association of the COVID-19 pandemic with the monthly incidence of A) Severe malnutrition (N = 59,365), B) Mild and moderate malnutrition (N = 75,995), and C) Overall malnutrition (N = 135,360) per 100,000 children younger than 18 years in France**. The black line shows the observed data. The green line shows the model estimates based on observed data using the segmented linear regression model. The orange dotted line shows the expected values of the scenario without the COVID-19 pandemic, using the same model. The green and orange shadings indicate the 95% confidence intervals. The dotted vertical line indicates the COVID-19 pandemic in March 2020. Abbreviation: CI, confidence interval.

We observed a 20.3% (95% CI, 10.7–29.9; p < 0.001) cumulative increase in the incidence of severe malnutrition associated with the COVID-19 pandemic (Fig. 2A, Table 2). In contrast, the incidence of mild and moderate malnutrition declined by 11.2% (95% CI, −18.7 to −3.6; p < 0.005), and overall malnutrition showed no significant change (cumulative change, 2.8%; 95% CI, −9.8 to 4.2; p = 0.44) (Fig. 2B and C, Table 2). Most substantial changes were observed among adolescents aged 11–17 years, who experienced a reduction in mild and moderate malnutrition but an increase in severe malnutrition (Table 2). A significant increase in the incidence of iron deficiency was also observed (eFigure S7 and eTable S3). In addition, among children hospitalized with scurvy during the post-COVID-19 period, the proportion of severe malnutrition was 22.6%, whereas autistic disorder and anorexia nervosa accounted for 5.8% and 5.0% of cases, respectively (eTable S4).

### Control outcomes

**Fig. 3:**
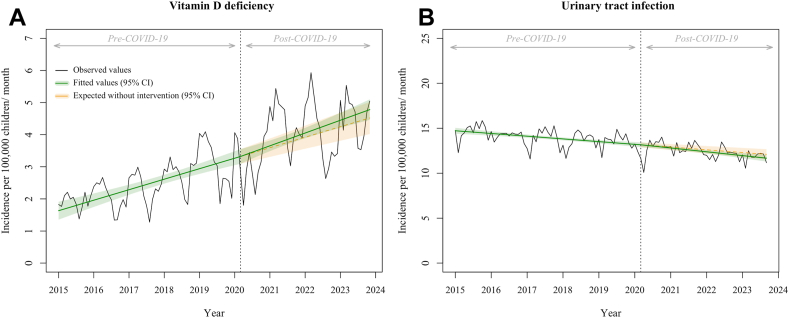
**Association of the COVID-19 pandemic with the monthly incidence of A) vitamin D deficiency (N = 44,003) and B) urinary tract infection per 100,000 children younger than 18 years (N = 184,374)**. The black line shows the observed data. The green line shows the model estimates based on observed data using the segmented linear regression model. The orange dotted line shows the expected values of the scenario without the COVID-19 pandemic, using the same model. The green and orange shadings indicate the 95% confidence intervals. The dotted vertical line indicates the COVID-19 pandemic in March 2020. Abbreviation: CI, confidence interval.

In contrast, the incidence of urinary tract infection did not significantly change following the COVID-19 pandemic (cumulative change, −1.7%; 95% CI, −4.9 to 1.4; p = 0.28). Similarly, the incidence of vitamin D deficiency showed a steady increase during the pre-COVID-19 period without significant change in trend following the COVID-19 pandemic (cumulative increase, 3.4%; 95% CI, −5.4 to 12.1; p = 0.45). These findings are detailed in Fig. 3 and Table 2. These results indicated that potential cointerventions—as a revision of the PMSI coding system—were minimal and did not bias the association between the COVID-19 and the changes in the incidence of scurvy and malnutrition.

### Association of scurvy with social deprivation and price inflation

Among children hospitalized for scurvy, we found a significant increase in the percentage of children covered by the State Universal Health Insurance in the post-COVID-19 period (mean, 27.6%; 95% CI, 22.8–32.5), compared to the pre-COVID-19 period (mean, 20.8%; 95% CI, 16.1–25.5), with a relative change of 32.9% (95% CI, 0.75–65.1, p = 0.045). Significant changes in the percentage of patients with Universal Health Insurance between the two periods were observed among the other clinical outcomes (Table 3), suggesting an increase in social deprivation since the COVID-19 pandemic.

**Table 3:** Percentage of children with universal health insurance for each outcome by study period, January 2015–November 2023.

Outcome	Mean percentage of patients with universal health insurance, % (95% CI)			
	Pre-COVID-19 period^a^	Post-COVID-19 period^b^	Relative change, % (95% CI)	p value^c^
Scurvy	20.8 (16.1–25.5)	27.6 (22.8–32.5)	+32.9 (0.75–65.1)	0.045
Severe malnutrition	12.9 (12.0–13.8)	14.8 (14.1–15.5)	+14.8 (6.0–23.6)	0.001
Mild malnutrition	14.1 (13.4–14.8)	17.1 (16.5–17.7)	+21.2 (14.8–27.6)	<0.001
Overall malnutrition	13.5 (12.8–14.2)	15.7 (15.3–16.2)	+16.3 (10.2–22.5)	<0.001
Iron deficiency	18.4 (17.6–19.2)	22.2 (21.5–22.8)	+20.5 (14.7–26.3)	<0.001
Vitamin D deficiency	16.2 (15.2–17.0)	19.3 (18.7–19.9)	+19.1 (10.0–30.9)	<0.001
Urinary tract infection	13.5 (13.1–13.9)	15.8 (15.6–16.1)	+17.0 (12.2–22.9)	<0.001

Abbreviation: CI, confidence interval.

a Pre-COVID-19 period from January 2015 to March 2020.

b Post-COVID-19 period from April 2020 to November 2023.

c Means were compared using the Student's t-test.

**Fig. 4:**
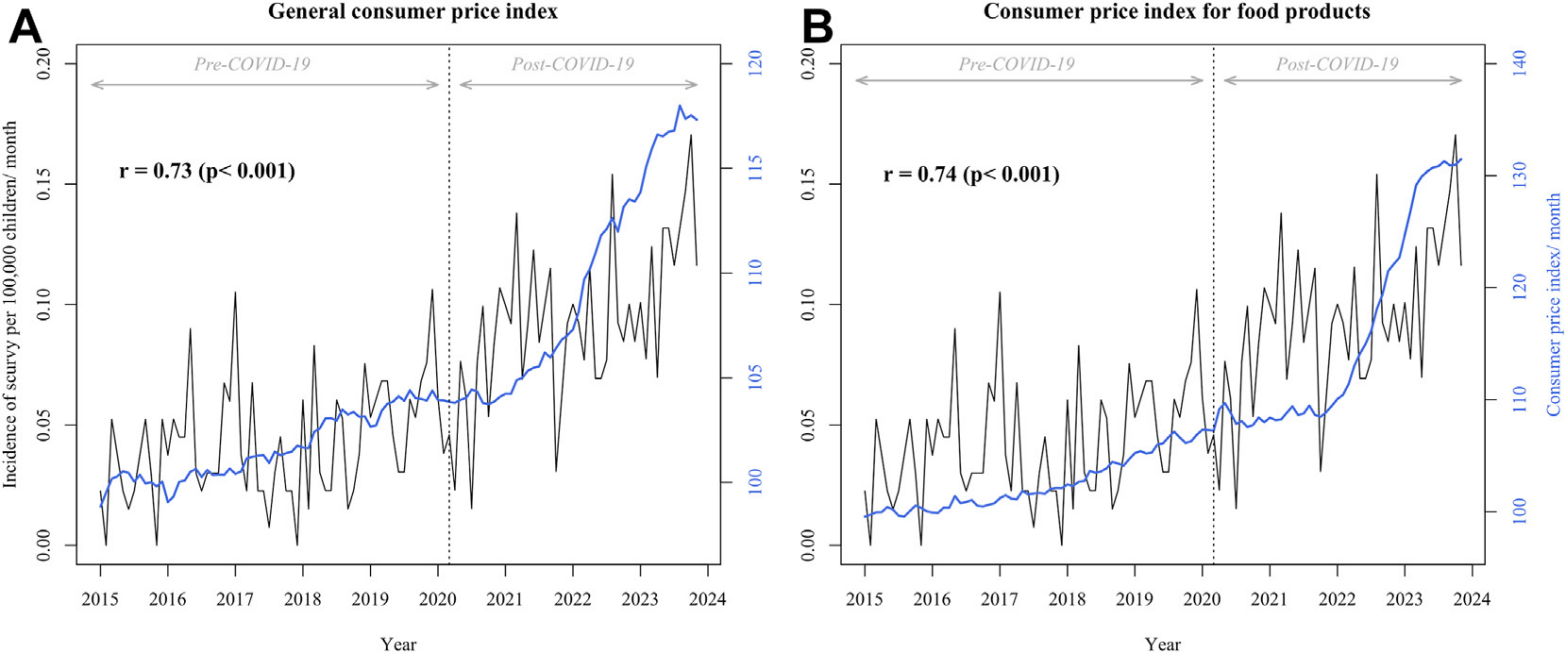
**Correlation of the monthly incidence of scurvy per 100,000 children younger than 18 years with A) the consumer price index and B) the consumer price index for food products**. r, Spearman correlation coefficient; the dotted vertical line indicates the COVID-19 pandemic in March 2020. Source: https://www.insee.fr/fr/statistiques/serie/001759963#Graphique.

The incidence of scurvy was significantly and positively correlated with the changes in the general consumer price index and the consumer price index for food products—used as proxies for price inflation—over the study period, with Spearman correlation coefficients of 0.73 (p < 0.001) and 0.74 (p < 0.001), respectively (Fig. 4). The consumer price index for food products was also significantly correlated with the incidence of severe malnutrition (coefficient, 0.80; p < 0.001) and iron deficiency (coefficient, 0.42; p < 0.001) (eFigure S8).

## Discussion

This time-series analysis of a comprehensive national cohort reveals a significant increase in the incidence of scurvy and severe malnutrition among French children in the aftermath of the COVID-19 pandemic. Previously considered rare in high-income countries such as France, these conditions have resurfaced, probably exacerbated by the socio-economic challenges intensified by the pandemic and associated crises. The resurgence of scurvy highlights the urgent need for targeted nutritional interventions and proactive actions by health professionals and policymakers to protect children's health and prevent future public health crises.

To our knowledge, this study is the first to assess scurvy in a paediatric population in Western Europe, specifically examining changes in the incidence of scurvy associated with the COVID-19 pandemic and subsequent crises. The resurgence of scurvy in high-income countries, including France, has been reported by healthcare professionals in several countries through case reports.^1^^,^^2^^,^^7^ Although there have been efforts to raise public awareness, including coverage by major outlets such as The Guardian,^23^ systematic data on scurvy incidence and long-term trends in high-income countries remain scarce. A recent study from the US reported a threefold increase in paediatric scurvy cases over five years (2016–2020), but did not assess the impact of the COVID-19 pandemic on this disease.^2^ Our findings, which report 888 cases in France over a nine-year period, highlight a critical public health issue that demands urgent attention from modern Western societies.

The post-pandemic period has intensified vulnerabilities in food security, driven by lasting effects of COVID-19 and major socio-geopolitical conflicts, such as the war in Ukraine. These events have set off a series of crises in health, energy, and inflation, all contributing to a global increase in food insecurity.^24^^,^^25^ In France, this led to an increase reliance on public and voluntary food aid, particularly among the self-employed, precariously employed and students.^24^^,^^25^ Our study demonstrates a significant increase in scurvy and severe malnutrition among children, linked to the escalation of food prices. A recent survey on the living conditions and aspirations of the French population highlighted a significant rise in food insecurity in France throughout 2022, with food inflation reaching 15% in January 2023, more than double the overall inflation rate.^26^ Nearly 75% of those affected attributed this to high food prices, with accessibility issues and social isolation further compounding this problem.^27^ Young adults below the age of 40 have been markedly affected, with 24% reporting inadequate nutrition, a rate significantly higher than older age groups,^26^ which extends to their children, who are becoming unwitting victims of the growing food insecurity crisis.

Our findings underscore a critical need to intensify food and social assistance programs, as current measures are not sufficient to curb the rising malnutrition and food insecurity rates, particularly among vulnerable populations, including children. Additionally, improved clinical training may be necessary to ensure early detection of scurvy and proactive screening of at-risk population. The resurgence of preventable nutritional deficiencies warrants prompt engagement with policymakers to advocate for data-driven, targeted policy responses, emphasizing structural interventions, that support families and children.

In this context, the identification of specific risk profiles within the paediatric population is crucial for the development of more targeted and effective interventions. In our study, scurvy was observed in all paediatric age groups (<4 years, 5–10 years, 11–17 years), with pre-adolescents and adolescents accounting for almost half of the cases. Children aged <4 years and 5–10 years each accounted for about 24% of cases, with an equal sex ratio, and only a small proportion of psychiatric disorders. This contrasts with US data for 2016–2020, which show a younger mean age of 2.15 years (standard deviation 4.78), a male predominance, and a very high proportion of autism.^2^ Following the COVID-19 pandemic, the incidence of scurvy increased significantly in children aged 5–10 years, with an increase of 200%, compared with only 10.2% and 7.3% in the other two age groups. Furthermore, sex-stratified analysis revealed that this post-pandemic surge in scurvy was predominantly observed in girls, which may reinforce previous data that emphasize gender disparities in food insecurity.^28^ These findings suggest that, in the current context, children aged 5–10 years—especially girls—are at increased risk of inadequate vitamin C intake.

Regarding severe malnutrition, we observed a 20.3% increase in severe malnutrition in the post-COVID-19 period, particularly among 11–17 years olds. Meanwhile, cases of mild and moderate malnutrition decreased, especially among children under 4 years and those aged 10–17 years. These trends suggest that patients with mild and moderate malnutrition may have progressed to more severe malnutrition after the pandemic. Similar to the findings in scurvy, sex-stratified analysis indicated that girls are more affected than boys, underscoring their heightened vulnerability in this context. The increase in severe malnutrition in a high-income country underlines the urgent need to re-evaluate care strategies, prevent loss of care, and improve efforts to re-engage those who may have been lost to follow-up.

Our study provides a comprehensive analysis of the epidemiology of scurvy in a European country over a nine-year peri-pandemic period. However, several limitations should be noted. First, due to the observational nature of the study, we cannot formally establish a causal relationship between the increase in scurvy incidence, malnutrition, and the COVID-19 pandemic. While several sensitivity analyses were conducted to assess the robustness of the findings, additional contributory factors may also play a role in the resurgence of scurvy beyond inflation. Further studies are required to assess the temporal association with other environmental changes which may have occurred during the study period. Second, our results may be influenced by concurrent interventions targeting the same outcomes, particularly changes in the PMSI coding system during the COVID-19 pandemic. Notably, the incidence of two control outcomes, vitamin D deficiency and urinary tract infection, remained stable over the study period, suggesting limited bias from these potential confounders. Third, as the PMSI database is hospital-based, our analysis only included inpatients, which likely underestimates the true incidence of vitamin deficiencies. Fourth, the PMSI database lacks detailed clinical and biological data at the individual level, with diagnoses based on ICD-10 codes from discharge summaries. However, the PMSI coding procedure undergoes internal quality control by the Medical Information Department and follows national guidelines by the Technical Agency for Hospital Information (ATIH),^16^ which limit the risk of coding errors. Fifth, our findings indicate a significant association between the post-pandemic increase in scurvy cases and changes in the consumer price index for food products, which is consistent with known risk factors for scurvy. However, our dataset does not allow us to examine the specific mechanisms or the isolated impacts of individual subsequent crises, including the COVID-19 pandemic, the war in Ukraine and subsequent energy crises. Sixth, we assessed low socio-economic status via eligibility to state universal health insurance, which increased across all patient groups during the study periods. Adjustments to eligibility criteria, the introduction of reforms, and reallocations between different aid programmes during the extended study period may have influenced the results. Nevertheless, despite potential reclassification effects, the scurvy patient group still experienced a relative increase of 32.7%, notably higher than in other groups, such as urinary tract infection group (17%) which likely represents the general paediatric population. This disproportionate increase underscores a pronounced level of social deprivation in the scurvy group in the post-pandemic period compared to both the other study and control groups.

Finally, as this study was conducted within a paediatric population in a single European country, our findings should be validated in other populations and regions to ensure broader generalizability. Considering that food insecurity and related socio-economic challenges are global, the resurgence of severe malnutrition and scurvy likely affects areas beyond France. Therefore, large-scale programs are needed to monitor these conditions in other countries.

### Conclusion

This time-series analysis of a national prospective cohort indicates a significant increase in hospitalised scurvy and severe malnutrition among French children in the post-COVID-19 era, with girls being particularly affected. These findings correlate with rising food prices, emphasizing the impact of socio-economic difficulties exacerbated by the pandemic and the concomitant crises. This concerning trend underscores the urgent need for targeting nutritional interventions and heightened awareness among health professionals and policymakers to address these emerging nutritional deficiencies, even in high-income countries. Ensuring adequate nutrition for all children is essential to prevent the resurgence of preventable diseases such as scurvy.

## Contributors

ZA, NO, and UM designed the study. MT, ZA and UM wrote the initial version of the manuscript. ZA, ZV, and NO performed the statistical analyses. MT, ZA, ZV, CD, AF, TI, FK, LO, NO and UM analysed and interpreted the data and drafted the article. All authors revised and approved the manuscript.

## Data sharing statement

Individual participant data required to reach aims in an approved proposal, after de-identification, will be made available to investigators whose proposed use of the data has been approved by the study's Executive Committee. Proposals may be submitted up to 36 months after article publication and should be directed to ulrich.meinzer@aphp.fr.

## Declaration of interests

We declare no competing interest.

## Appendix A Supplementary data

eFigures and eTables
