# Supplementary material for: Scurvy incidence trend among children hospitalised in France, 2015–2023: a population-based interrupted time-series analysis
Source: Lancet Reg Health Eur. 2024 Dec 6;49:101159. doi: 10.1016/j.lanepe.2024.101159 (PMC11667167; doi:10.1016/j.lanepe.2024.101159)
Supplement: eFigures and eTables [file mmc1.docx]

**Supplementary Materials**

Scurvy incidence trend among children hospitalised in France, 2015-2023: a population-based interrupted time-series analysis

**eFigure S1.** Association of the COVID-19 pandemic with the monthly incidence of Scurvy per 100 000 children younger than 18 years, by sex.

**eFigure S2**. Correlograms and residuals analysis of the main segmented linear regression model for the monthly incidence of scurvy per 100 000 children.

**eTable S1.** Characteristics of the main segmented linear regression model for the association of the COVID-19 pandemic with the incidence of scurvy per 100 000 children.

**eTable S2**. Sensitivity analyses for the association between the COVID-19 pandemic and the incidence of scurvy in France, January 2015 to November 2023.

**eFigure S3**. Sensitivity analysis including one-year transitional period for the association of the COVID-19 pandemic with the monthly incidence of Scurvy per 100 000 children younger than 18 years (N=888).

**eFigure S4**. Correlograms and residuals analysis of the segmented linear regression model including harmonic terms with 6- and 12-month periods for the monthly incidence of scurvy per 100 000 children.

**eFigure S5**. Correlograms and residuals analysis of the segmented linear regression model including harmonic terms with 3-, 6- and 12-month periods for the monthly incidence of scurvy per 100 000 children.

**eFigure S6**. Correlograms and residuals analysis of the segmented linear regression model with one-year transitional period for the monthly incidence of scurvy per 100 000 children.

**eFigure S7.** Association of the COVID-19 pandemic with the monthly incidence of iron deficiency per 100 000 children younger than 18 years (N=46 112).

**eTable S3.** Association between the COVID-19 pandemic and the incidence of iron deficiency in France, January 2015 to November 2023 (N= 46 112).

**eTable S4.** Most frequent diseases found in children hospitalized with scurvy in France, January 2015 to November 2023 (N=888).

**eFigure S8.** Correlation of the consumer price index for food products with the monthly incidence of A) severe malnutrition and B) iron deficiency per 100 000 children younger than 18 years.

**eFigure S1. Association of the COVID-19 pandemic with the monthly incidence of Scurvy per 100 000 children younger than 18 years, by sex.**

**
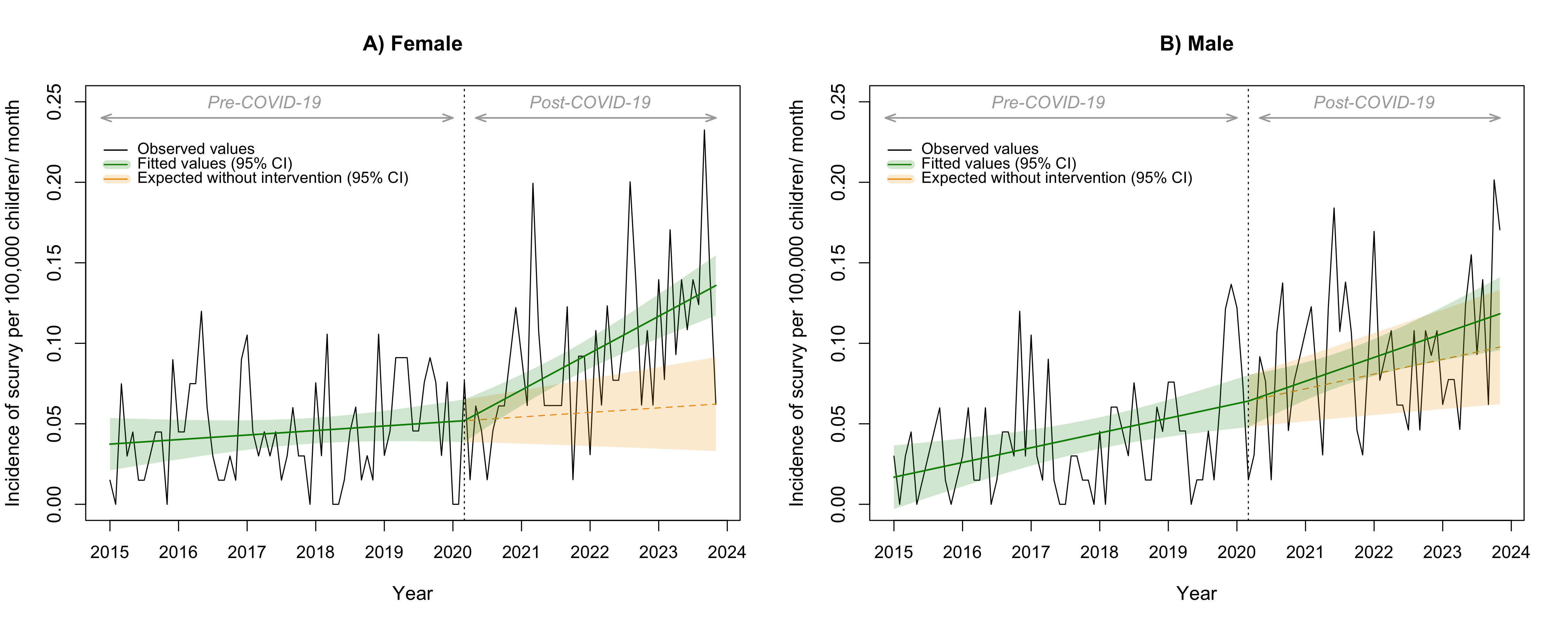
**

The black line shows the observed data. The green line shows the model estimates based on observed data using the segmented linear regression model. The orange dotted line shows the expected values of the scenario without the COVID-19 pandemic, using the same model. The green and orange shadings indicate the 95% confidence intervals. The dotted vertical line indicates the COVID-19 pandemic in March 2020.

**eFigure S2. Correlograms and residuals analysis of the main segmented linear regression model for the monthly incidence of scurvy per 100 000 children.**

To assess the quality of the segmented linear regression model, we used correlograms (autocorrelation and partial autocorrelation functions which measure the linear relationship between lagged values of a time series) and residuals analysis. Inspection of the correlograms relies on identifying remaining autocorrelation or seasonal pattern of the residuals. The significance of any remaining autocorrelation or seasonality is defined by a correlation higher than +1.96 standard error or lower than -1.96 standard error for each lag of the time series. We checked whether the residuals of the models were normally distributed and had a constant variance over time. The correlograms were satisfactory (no remaining autocorrelation nor seasonal pattern of the residuals)”

ACF, autocorrelation function.

**eTable S1. Characteristics of the main segmented linear regression model for the association of the COVID-19 pandemic with the incidence of scurvy per 100 000 children.**

| **Model Parameters** | **Segmented linear regression model** |
| --- | --- |
|  |  |
| **Dependent variable** | monthly incidence of scurvy per 100 000 children aged < 18 years |
| **Time unit** | 1 month |
| **Independent variables** |  |
| Temporal trend | time set as a continuous variable with the number of months since the start of the study |
| Seasonality | removed using additive model |
| Autocorrelation | autoregressive–moving-average (ARMA) term |
| COVID-19 | “ramp” function for progressive change in trend after March 2020 |
| **Time points** |  |
| Pre-COVID-19 | 63 (months) |
| Post-COVID-19 | 44 (months) |
| **Time series length** | 107 (months) |
| **Fitted values** | estimated scurvy incidence based on the independent variables |
| **Counterfactual values** | estimated scurvy incidence based on the independent variables with intervention term set to zero |
| **Intervention impact calculation** | relative change in scurvy incidence between fitted and counterfactual values in the post-COVID-19 period |
| **Model validity checking^a^** | visual inspection of the correlograms (autocorrelation and partial autocorrelation functions) and residuals analysis |

^a^ Details in eFigure S1.

**eTable S2. Sensitivity analyses for the association between the COVID-19 pandemic and the incidence of scurvy in France, January 2015 to November 2023.**

| **Outcome** | **Monthly change in trend in the pre-COVID-19 period**^a^**, % (95% CI)** | **Change of slope following COVID-19**^b^**, % (95% CI)** | **Cumulative change in the post-COVID-19 period, % (95% CI)** | ***P* value for cumulative change** |
| --- | --- | --- | --- | --- |
| **Incidence of scurvy per 100 000 children**^c^ |  |  |  |  |
| Segmented linear regression model with  trigonometric function (6-12 m)^d^ | 0.05 (0.02 to 0.08) | 1.6 (0.63 to 2.6) | 34.2 (13.4 to 55.0) | 0.002 |
| Segmented linear regression model with  trigonometric function (3-6-12 m)^d^ | 0.05 (0.02 to 0.08) | 1.7 (0.66 to 2.7) | 34.7 (13.9 to 55.6) | 0.002 |
| Segmented linear regression model with 1-year transitional period^e^ | 0.04 (0.01 to 0.08) | 1.5 (0.53 to 2.5) | 47.5 (16.8 to 78.2) | 0.004 |

^a^ pre-COVID-19 period from January 2015 to March 2020.

^b^ COVID-19 pandemic started in March 2020.

^c^ Monthly incidence expressed as the number of cases per 100 000 children.

^d^ post-COVID-19 period from April 2020 to November 2023.

^e^ post-COVID-19 period from April 2021 to November 2023.

**eFigure S3. Sensitivity analysis including one-year transitional period for the association of the COVID-19 pandemic with the monthly incidence of Scurvy per 100 000 children younger than 18 years (N=888).**


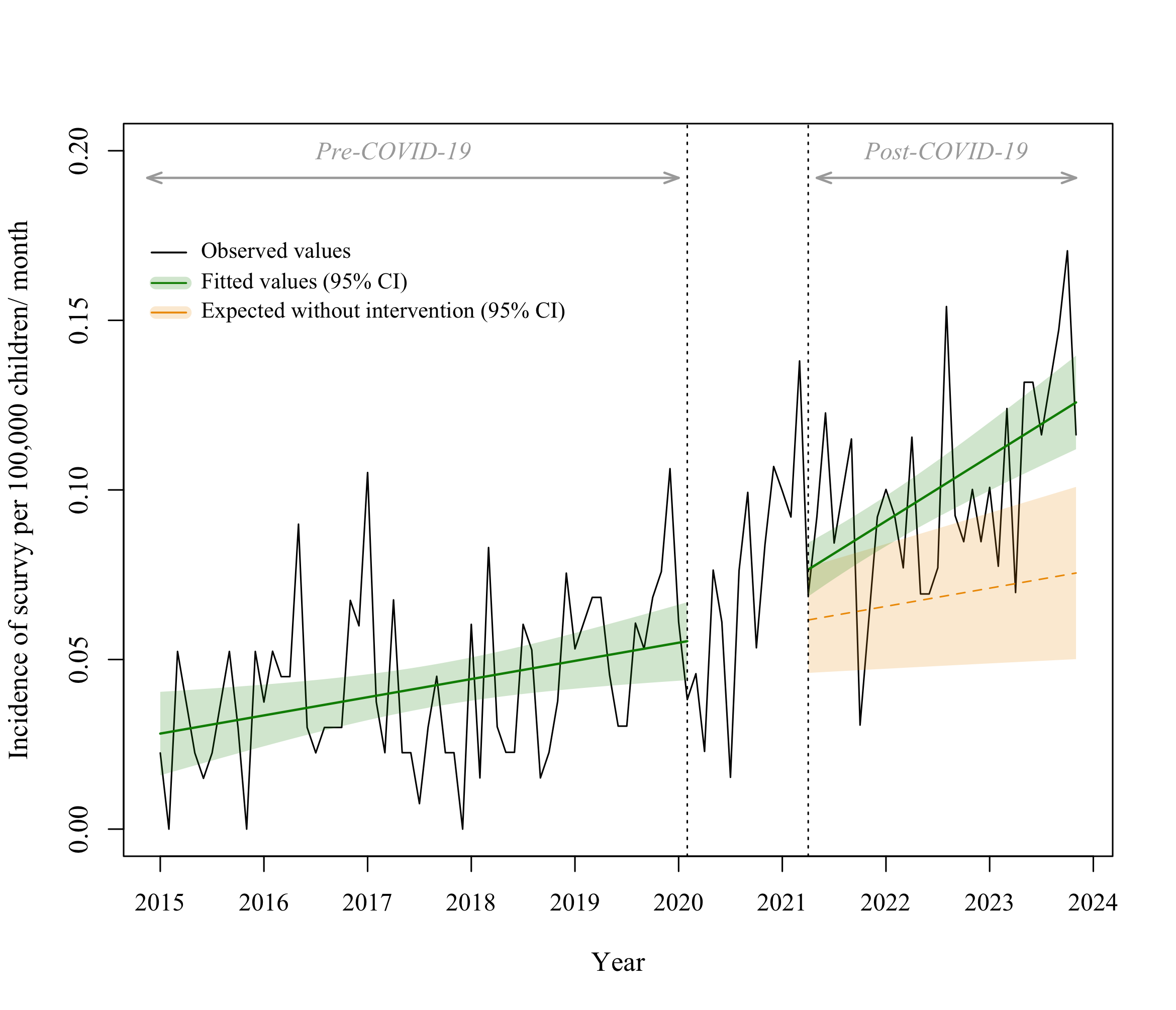


The black line shows the observed data. The green line shows the model estimates based on observed data using the segmented linear regression model. The orange dotted line shows the expected values of the scenario without the COVID-19 pandemic, using the same model. The green and orange shadings indicate the 95% confidence intervals. The dotted vertical lines indicate the COVID-19 pandemic in March 2020 and the release of the lockdowns and other non-pharmaceutical interventions related to the COVID-19 pandemic in April 2021. The transitional period is from March 2020 to March 2021. The change in the incidence of scurvy was therefore analysed from April 2021 to November 2023.

**eFigure S4. Correlograms and residuals analysis of the segmented linear regression model including harmonic terms with 6- and 12-month periods for the monthly incidence of scurvy per 100 000 children.**

To assess the quality of the segmented linear regression model, we used correlograms (autocorrelation and partial autocorrelation functions which measure the linear relationship between lagged values of a time series) and residuals analysis. Inspection of the correlograms relies on identifying remaining autocorrelation or seasonal pattern of the residuals. The significance of any remaining autocorrelation or seasonality is defined by a correlation higher than +1.96 standard error or lower than -1.96 standard error for each lag of the time series. We checked whether the residuals of the models were normally distributed and had a constant variance over time. The correlograms were satisfactory (no remaining autocorrelation nor seasonal pattern of the residuals)”

ACF, autocorrelation function.

**eFigure S5. Correlograms and residuals analysis of the segmented linear regression model including harmonic terms with 3-, 6- and 12-month periods for the monthly incidence of scurvy per 100 000 children.**

To assess the quality of the segmented linear regression model, we used correlograms (autocorrelation and partial autocorrelation functions which measure the linear relationship between lagged values of a time series) and residuals analysis. Inspection of the correlograms relies on identifying remaining autocorrelation or seasonal pattern of the residuals. The significance of any remaining autocorrelation or seasonality is defined by a correlation higher than +1.96 standard error or lower than -1.96 standard error for each lag of the time series. We checked whether the residuals of the models were normally distributed and had a constant variance over time. The correlograms were satisfactory (no remaining autocorrelation nor seasonal pattern of the residuals)”

ACF, autocorrelation function.

**eFigure S6. Correlograms and residuals analysis of the segmented linear regression model with one-year transitional period for the monthly incidence of scurvy per 100 000 children.**

To assess the quality of the segmented linear regression model, we used correlograms (autocorrelation and partial autocorrelation functions which measure the linear relationship between lagged values of a time series) and residuals analysis. Inspection of the correlograms relies on identifying remaining autocorrelation or seasonal pattern of the residuals. The significance of any remaining autocorrelation or seasonality is defined by a correlation higher than +1.96 standard error or lower than -1.96 standard error for each lag of the time series. We checked whether the residuals of the models were normally distributed and had a constant variance over time. The correlograms were satisfactory (no remaining autocorrelation nor seasonal pattern of the residuals)”

ACF, autocorrelation function.

**eFigure S7. Association of the COVID-19 pandemic with the monthly incidence of iron deficiency per 100 000 children younger than 18 years (N=46 112).**


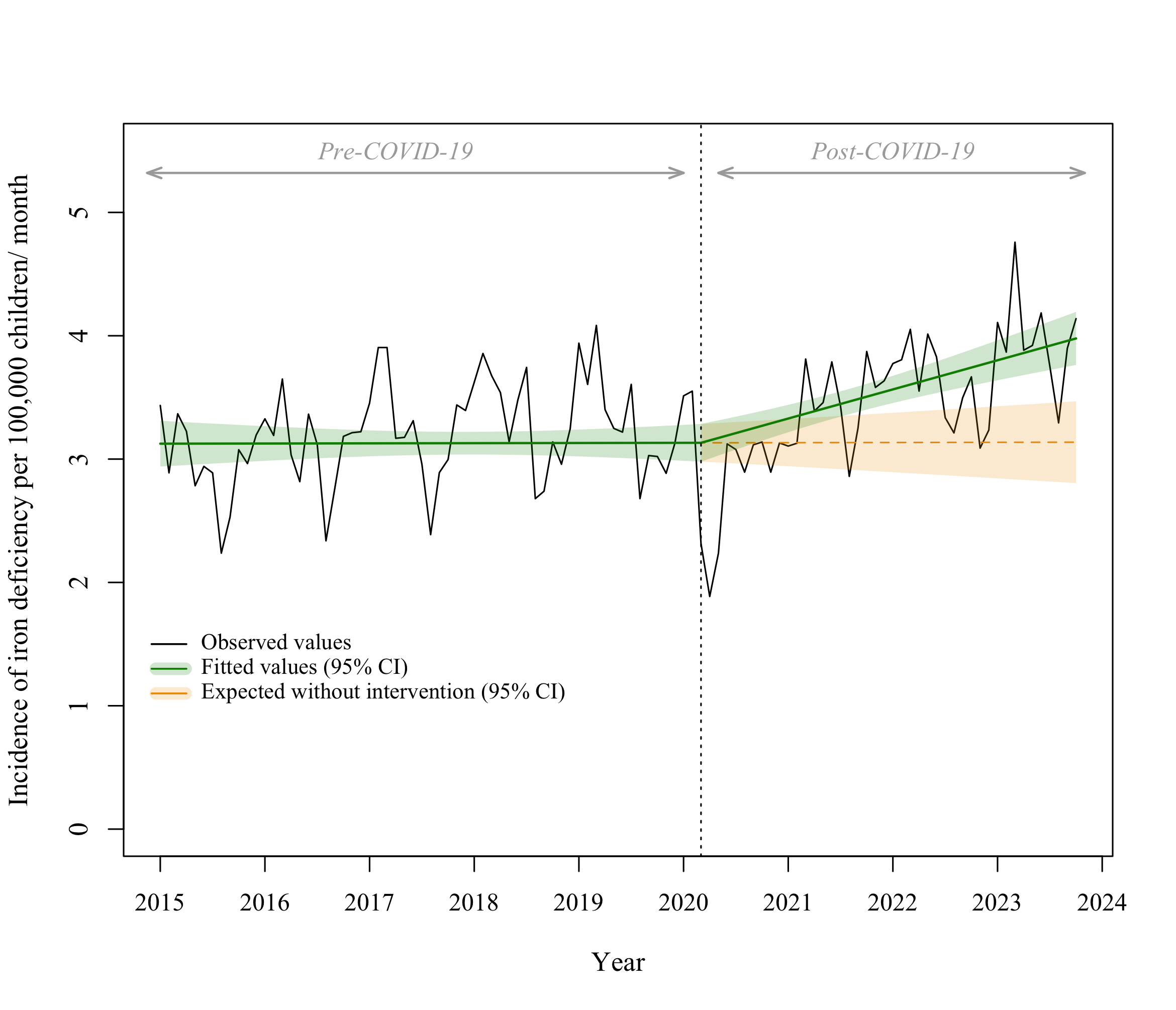


The black line shows the observed data. The green line shows the model estimates based on observed data using the segmented linear regression model. The orange dotted line shows the expected values of the scenario without the COVID-19 pandemic, using the same model. The green and orange shadings indicate the 95% confidence intervals. The dotted vertical lines indicate the COVID-19 pandemic in March 2020 and the release of the lockdowns and other non-pharmaceutical interventions related to the COVID-19 pandemic in April 2021. The transitional period is from March 2020 to March 2021. The change in the incidence of scurvy was therefore analysed from April 2021 to November 2023.

**eTable S3. Association between the COVID-19 pandemic and the incidence of iron deficiency in France, January 2015 to November 2023 (N=** **46 112).**

| **Outcome** | **Monthly change in trend in the pre-COVID-19 period**^a^**, % (95% CI)** | **Change of slope following COVID-19**^b^**, % (95% CI)** | **Cumulative change in the post-COVID-19 period, % (95% CI)** | ***P* value for cumulative change** |
| --- | --- | --- | --- | --- |
| **Incidence of iron deficiency**^c^ | 0.01 (-0.44 to 0.47) | 0.62 (0.29 to 0.96) | 13.7 (6.4 to 21.0) | < 0.001 |

^a^ pre-COVID-19 period from January 2015 to March 2020.

^b^ COVID-19 pandemic started in March 2020.

^c^ Monthly incidence expressed as the number of cases per 100 000 children.

**eTable S4. Most frequent diseases found in children hospitalized with scurvy in France, January 2015 to November 2023 (N=888).**

|  |  | **Children, n (%)** | | | |
| --- | --- | --- | --- | --- | --- |
| **ICD-10 code** | **Diagnosis** | **Pre-COVID period,^a^ N=352** | **Post-COVID-19 period,^b^ N=536** | **Total,**  **N=888** | **Rank** |
| E55, E550, E559 | Vitamin D deficiency | 101 (28.7) | 184 (34.3) | 285 (32.1) | 1 |
| E43, E44, E440, E441, E46 | Overall malnutrition | 87 (24.7) | 160 (29.9) | 247 (27.8) | 2 |
| E43 | Severe malnutrition | 43 (12.2) | 121 (22.6) | 164 (18.5) | 3 |
| E53, E530, E531, E538, E539 | Deficiency of other specified B group vitamins | 38 (10.8) | 93 (17.4) | 131 (14.8) | 4 |
| E44, E440, E441, E46 | Mild and moderate malnutrition | 49 (13.9) | 53 (9.9) | 102 (11.5) | 5 |
| Z608, Z609, Z59, Z591, Z595, Z596, Z597, Z598, Z599, Z590, Z60, Z6030, Z608 | Socio-economic and environmental difficulties | 17 (4.8) | 29 (5.4) | 46 (5.2) | 6 |
| E611, D509 | Iron deficiency and iron deficiency anaemia | 16 (4.5) | 29 (5.4) | 45 (5.1) | 7 |
| F500 | Anorexia nervosa | 16 (4.5) | 27 (5.0) | 43 (4.8) | 8 |
| F84, F840, F841, F841, F8411, F842, F848, F849 | Autistic disorder | 7 (2.0) | 31 (5.8) | 38 (4.3) | 9 |
| R633, F982 | Feeding difficulties and other feeding disorders of infancy and childhood | 9 (2.6) | 12 (2.2) | 21 (2.4) | 10 |

Data are presented as numbers (%).

^a^ pre-COVID-19 period from January 2015 to March 2020.

^b^ post-COVID-19 period from April 2020 to November 2023.

**eFigure S8. Correlation of the consumer price index for food products with the monthly incidence of A) severe malnutrition and B) iron deficiency per 100 000 children younger than 18 years.**

**
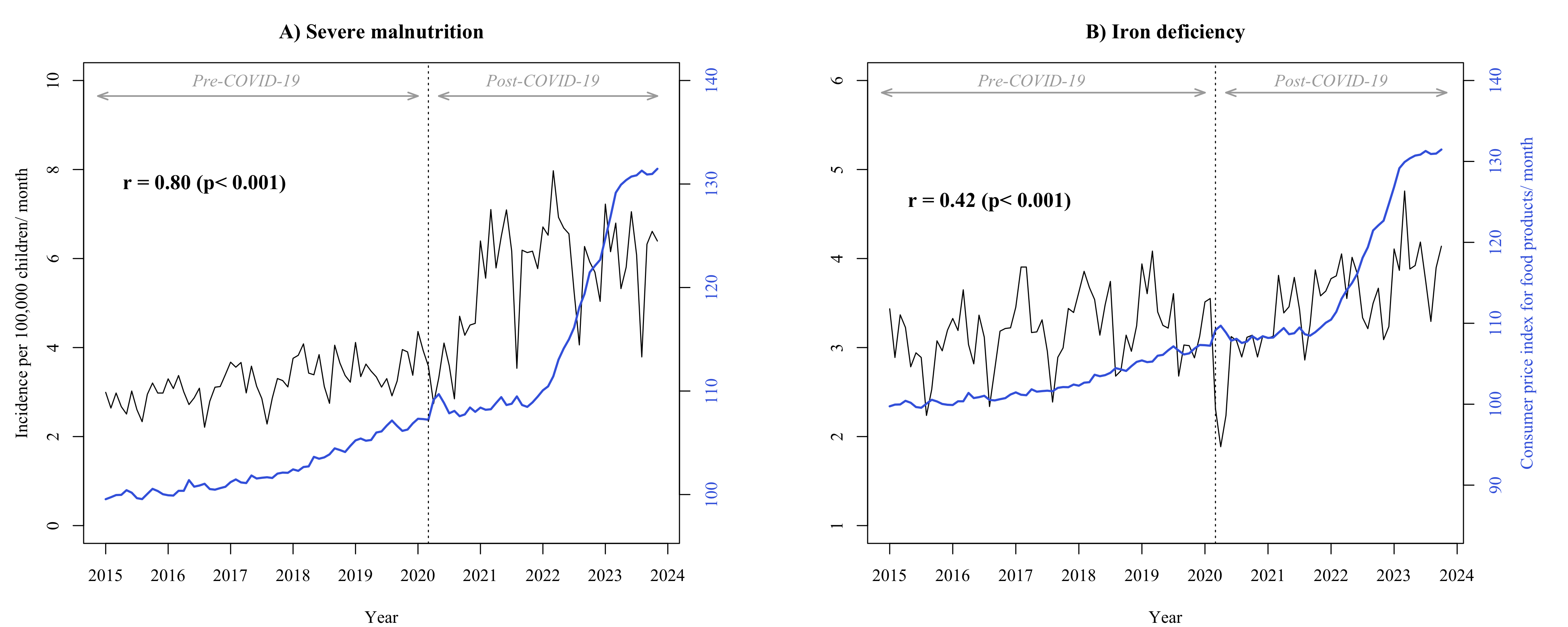
**

r, Spearman correlation coefficient; the dotted vertical line indicates the COVID-19 pandemic in March 2020.

Source: <https://www.insee.fr/fr/statistiques/serie/001759963#Graphique>
